# The impact of COVID‐19 on the management of neuroendocrine tumors (NETS): An international NET CONNECT survey of NET patients and healthcare professionals treating net patients

**DOI:** 10.1111/jne.13196

**Published:** 2022-09-07

**Authors:** Mauro Cives, Jorge Hernando, Angela Lamarca, Catherine Bouvier, Martyn Caplin, Marianne Pavel

**Affiliations:** ^1^ Department of Biomedical Sciences and Human Oncology University of Bari “Aldo Moro” Bari Italy; ^2^ Department of Medical Oncology Vall d'Hebron University Hospital, Vall d'Hebron Institute of Oncology Barcelona Spain; ^3^ Department of Medical Oncology The Christie NHS Foundation, Manchester; Division of Cancer Sciences, University of Manchester Manchester UK; ^4^ International Neuroendocrine Cancer Alliance (INCA) Boston Massachusetts USA; ^5^ Neuroendocrine Tumor Unit Royal Free Hospital London UK; ^6^ Department of Medicine 1, Endocrinology Friedrich Alexander Universität Erlangen‐Nürnberg Erlangen Germany

**Keywords:** carcinoids, SARS‐CoV‐2, telemedicine, vaccination

## Abstract

The COVID‐19 outbreak has added complexity in the management of patients with neuroendocrine tumors (NETs). Little information is currently available regarding the real impact of the pandemic in current practice. The present study aimed to capture patients' and healthcare professionals' experiences on how the NET management has changed during the pandemic and how it should be modified in a foreseeable post‐pandemic environment. Physicians and nurses working in ENETS Centers of Excellence or other hospitals with high volume of NET patients (*n* = 48), as well as NET patients residing worldwide (*n* = 353), were asked to respond to two online anonymous surveys addressing different aspects of NET care. Deferred diagnoses, delayed surveillance procedures and postponed elective surgeries were among the main negative consequences of the COVID‐19 outbreak according to 40%, 54% and 46% of healthcare professionals (HPs) respectively. Somatostatin analogs were increasingly used as bridging strategy for delaying surgery based on the views of 31% of HPs and were self‐injected or delivered by home care services more frequently than before the initiation of the pandemic (53% of patients during the pandemic vs. 44% before the pandemic). Multidisciplinary tumor boards kept their usual schedule according to 58% of HPs, but were held virtually in the 77% of cases. The contact with healthcare professionals was maintained by remote methods more often than in the past (69% of patients), but only 34% of patients (59% among subjects < 41 years) would prefer telemedicine to face‐to‐face consultations in the future. New health policy measures should guarantee the highest standard of treatment to NET patients, regardless of the trajectory followed by the COVID‐19 pandemic in the next months. Pros and cons of telemedicine should be carefully weighted before systematic implementation.

## Graphical abstract

The COVID‐19 outbreak has added complexity in the management of patients with NETs. This study aims to capture patients' and healthcare professionals' experiences on how the NET management has changed during the pandemic and how it should be modified in a foreseeable post‐pandemic environment. New health policy measures must guarantee the highest standard of treatment to NET patients, regardless of the trajectory followed by the COVID‐19 pandemic.

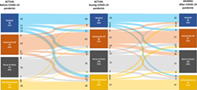

## 1 INTRODUCTION

Coronavirus disease 2019 (COVID‐19) is an infectious disease caused by a novel enveloped RNA betacoronavirus named severe acute respiratory syndrome coronavirus 2 (SARS‐CoV‐2).^1^ The first cluster of COVID‐19 pneumonia cases emerged in Wuhan, China in early December 2019 and rapidly spread all over the world, leading the World Health Organization (WHO) to declare the pandemic status on March 11, 2020.^1^, ^2^ Thus far, more than 418 million total confirmed cases of COVID‐19 have been recorded globally, resulting in approximately 5,800,000 SARS‐CoV‐2‐related deaths.^3^

Patients with cancer are more susceptible to SARS‐CoV‐2 infections,^4^ tend to develop more severe forms of COVID‐19^5^ and show a higher risk of COVID‐19 mortality with respect to the general population,^6^, ^7^, ^8^, ^9^, ^10^ probably as the consequence of advanced age, coexisting chronic comorbidities, cancer‐related and drug‐related immunosuppression. Although vaccination against COVID‐19 represents a key strategy in protecting vulnerable populations, patients with cancer who develop breakthrough infection despite full vaccination remain at risk of severe outcomes, with a 30‐day mortality of approximately 15%.^11^ Moreover, permanent treatment discontinuation or dose/regimen adjustments have been recorded in more than 50% of patients on systemic anti‐cancer therapy after SARS‐CoV‐2 infection, and treatment discontinuation has been identified as an independent predictor of death.^12^

Neuroendocrine tumors (NETs) are heterogeneous malignancies arising from the diffuse neuroendocrine system.^13^ Although NETs may develop in almost any organ, they predominate in the gastroenteropancreatic tract and bronchopulmonary system, with survival durations primarily dependent on primary tumor site, grade and stage.^13^ Based on a preliminary analysis of an international registry focusing on NET patients who tested positive for SARS‐CoV‐2, temporary or permanent antitumor treatment discontinuation was recorded in approximately one fourth of the patients, with a COVID‐19‐related mortality of 8%.^14^ Moreover, a substantial disruption of NET services including deferred surgeries, delayed peptide receptor radionuclide therapy initiation, postponed follow‐up examinations and canceled multidisciplinary activities has been reported as result of the COVID‐19 pandemic among different European countries.^15^, ^16^, ^17^

In the present study, we aimed to capture updated information with respect to the impact of COVID‐19 pandemic on the clinical management of NETs. In particular, both patients and healthcare professionals were invited to share their experiences and perspectives on how treatment decisions have been and will be modified during the COVID‐19 pandemic.

## 2 MATERIALS AND METHODS

### 2.1 Survey design

The NET CONNECT COVID taskforce (CM, HJ, LA, BC, CM and PM) designed two online surveys addressing different aspects of NET care including the role of telemedicine (see Supporting information, Appendix S1). One survey was dedicated to healthcare professionals (HP) working in European Neuroendocrine Tumor Society (ENETS)‐certified Centers of Excellence (CoE) or institutions with high volume of NET patients, whereas the other was dedicated to NET patients residing worldwide. An invitation to participate in the HP survey was sent by the SurveyMonkey platform (https://it.surveymonkey.com) to the lead of the ENETS CoE or a representative of institutions with a specialist reputation in neuroendocrine tumors. The patient survey, hosted on the Qualtrics platform (https://www.qualtrics.com/uk), was available on the International Neuroendocrine Cancer Alliance (INCA) website (https://incalliance.org), as well as on the websites of national patient associations, and was promoted through social media. The patient survey was available in English, French, Spanish and Italian, whereas the HP survey was available only in English. Participation was voluntary and anonymous for both surveys. Responses were collected between March 24, 2021 and August 19, 2021 for the HP survey and between and May 10, 2021 and August 31, 2021 for the patient survey. The study complied with the Declaration of Helsinki. No ethical committee approval was required.

### 2.2 Survey measures

Sociodemographic, background variables and three key outcomes of interest (clinical management changes, role of COVID‐19 vaccination, role of telemedicine) were collected in both the HP and patient surveys (see Supporting information, Appendix S1). Either yes/no questions, single or multiple choice questions or five‐point Likert scale questions (strongly agree to strongly disagree) were included in the surveys. Responses from HPs were stratified according to nationality, patient volume and specialties, whereas responses from patients were stratified according to nationality, age and active treatment/surveillance. Differences among such subgroups were reported only when statistically significant.

### 2.3 Statistical analysis

Descriptive data were presented as median (interquartile range) or the mean (± SD), and proportions were expressed as percentages. Chi‐squared or Fisher's exact tests were used to compare categorical variables. All tests were two‐sided and statistical significance was declared at *p* < .05. Statistical analysis was conducted using Prism, version 5.0 (GraphPad Software Inc., San Diego, CA, USA).

## 3 RESULTS

### 3.1 Demographic and baseline characteristics of participants

In total, 81 HPs were invited to participate in our study, and 48 of them (oncologists, *n* = 12; endocrinologists, *n* = 11; gastroenterologists, *n* = 8; surgeons, *n* = 5; nuclear medicine physicians, *n* = 2; internists, *n* = 1; specialist nurses, *n* = 9) completed the survey (overall response rate: 59%) (Table 1). HPs worked in 13 countries, of which 42 (88%) were from Europe, Overall, there were 25 (52%) male participants; 36 (75%) were over 40 years of age and 32 (67%) had > 10 years of experience in the respective discipline. The majority of the respondents (52%) worked in centers providing care for > 500 NET patients. Notably, among the 62 CoE certified by ENETS worldwide (all of which were invited to reply to this survey), 30 (48%) responded to the survey. The most common place of work was University hospitals (79%). In 58% of cases, respondents did not have exact information on the number of NET patients who tested positive for SARS‐CoV‐2 at their institutions.

**TABLE 1:** Participant demographic and baseline characteristics

	HPs (*n* = 48), *n* (%)	Patients (*n* = 353), *n* (%)
Gender		
Female	23 (48)	236 (67)
Male	25 (52)	117 (33)
Age (years)		
< 40	12 (25)	35 (10)
41–50	15 (31)	66 (19)
51–60	17 (35)	121 (34)
> 60	4 (8)	131 (37)
Country		
UK	18 (38)	52 (15)
Germany	5 (10)	6 (2)
France	5 (10)	22 (6)
USA	5 (10)	29 (8)
Italy	3 (6)	42 (12)
Netherlands	3 (6)	0
Spain	2 (4)	48 (14)
Ireland	1 (2)	29 (8)
Australia	0	67 (19)
Canada	0	36 (10)
Others	6 (13)	22 (6)
Tested positive for SARS‐CoV‐2		
Yes	5 (10)	25 (7)
No	43 (90)	327 (93)
Primary place of work		
University hospital	38 (79)	–
Public general hospital	5 (10)	–
Comprehensive cancer center	5 (10)	–
Specialty		
Oncology	12 (25)	–
Endocrinology	11 (23)	–
Gastroenterology	6 (13)	–
Surgery	5 (10)	–
Nursing	9 (19)	–
Others	5 (10)	
Years of experience		
< 5	4 (8)	–
6–10	12 (25)	–
11–20	21 (44)	–
> 20	11 (23)	–
Institutional volume of NETs		
51–100	4 (8)	–
101–250	9 (19)	–
251–500	10 (21)	–
> 500	25 (52)	–
Years since NET diagnosis		
< 2	–	69 (19)
2–5	–	149 (42)
6–10	–	73 (21)
> 10	–	62 (18)
Active treatment		
Yes	–	299 (85)
No	–	54 (15)
Member of a NET patient association/advocacy group		
Yes	–	224 (63)
No	–	129 (37)

Abbreviation: NET, neuroendocrine tumor.

In addition, 353 participants from 23 countries responded to the patient survey (Table 1). Most of the respondents were females (67%) and the age was over 50 years in 71% of them. The diagnosis of NET dated back at least 2 years in 81% of respondents, and 85% of them stated to be on active treatment. All healthcare providers and patients answered the survey completely.

### 3.2 COVID‐19‐induced changes in the management of NETs: the HP perspective

When interrogated on the main sources of information used for clinical decisions in the NET management during the pandemic, HPs reported to have received guidance from professional societies (including ESMO, ENETS and NANETS) in 73% of cases. Interestingly, this percentage increased up to 95% among respondents working in centers providing care for > 500 NET patients, in contrast to 56% among those working in lower volume institutions (*p* = .003). Local country based specific recommendations were available only in 38% of cases, and were followed in all cases when known by HPs.

**FIGURE 1:**
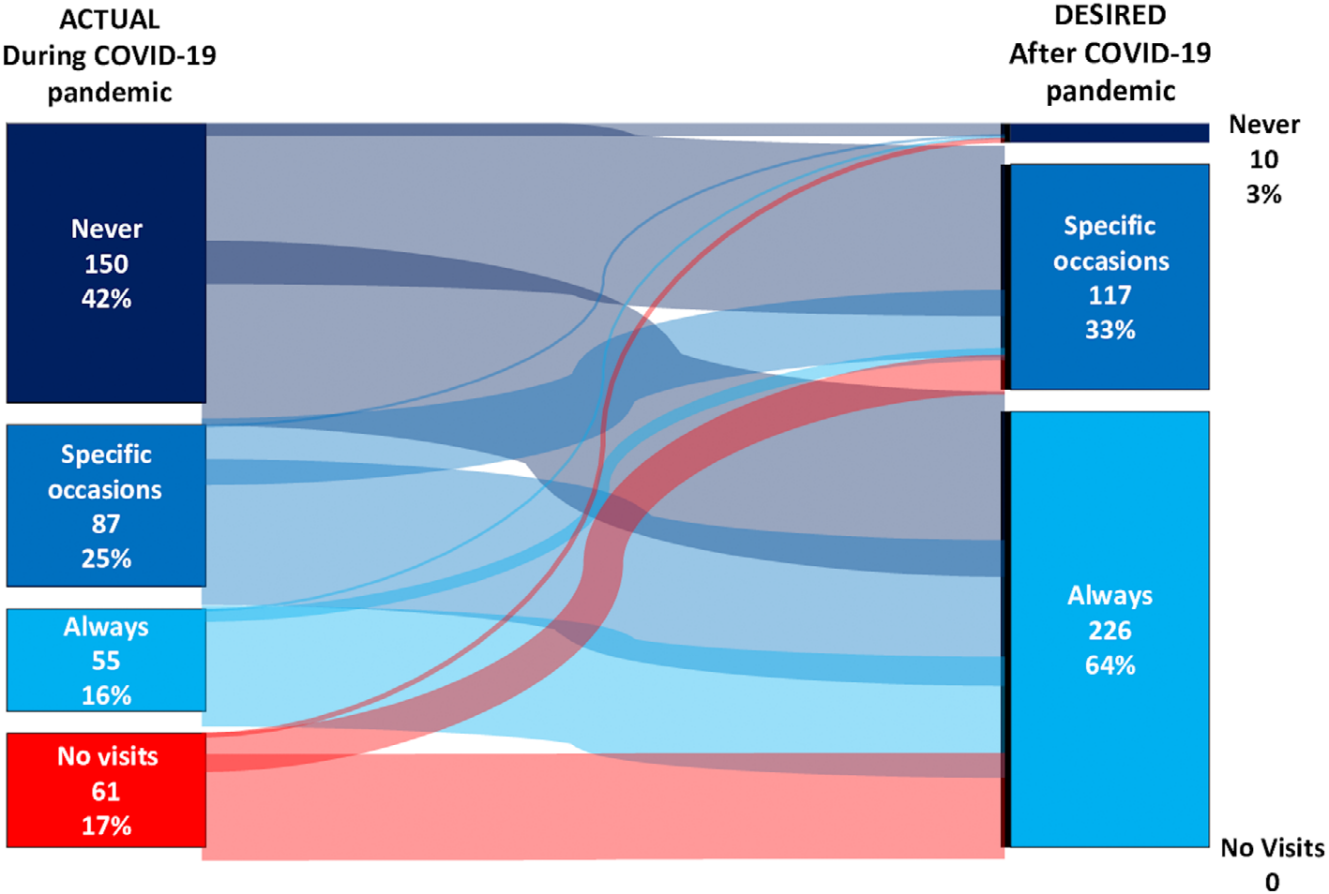
Family attendance during face‐to‐face consultations: experiences and perspectives. Sankey diagrams show changes in actual and desired frequency of family attendance to face‐to‐face consultations among patients. No differences were observed by stratifying factors (nationality, age, being on active treatment/surveillance)

According to 48% of HP respondents, the pandemic negatively affected their relationship with NET patients, with contact mainly maintained via online consultations (63%) or mails/phone calls (27%). This phenomenon could not be ascribed to the lack of personal protective equipment (available in 94% of cases). When face‐to‐face visits were performed, family members were allowed to accompany the patient only on selected occasions (i.e., when deemed necessary by the treating team or upon explicit patient request) according to 75% of responses. Patients who were required to attend the consultations on their own appeared to understand such a need according to the perception of 85% of HPs. Before face‐to‐face consultations, patients were screened for COVID‐related symptoms in 77% of cases, whereas a negative SARS‐CoV‐2 test was required only according to 6% of respondents. For future face‐to‐face consultations during the pandemic, 38% and 62% of the HPs wished family members to be allowed always or on specific occasions, respectively (Figure 1).

Among the main patient worries perceived by clinicians were the risk of COVID‐19‐related complications (96%), difficulties in the management of their disease (73%) or their oncological medications (85%). HPs advised on general health measures in 79% of cases during the consultations. Among the multiple healthcare disruptions caused by the COVID‐19 pandemic, HPs identified a deteriorated capacity to assess patients (67%) and difficulties in the diagnostic pathway (40%) as the main criticisms.

A negative impact of the pandemic on NET treatment was reported only by 15% of the respondents. Nevertheless, an increased adoption of watch and wait strategies was reported by one third of HPs, with surgical interventions or surveillance exams being postponed according to 46% and 54% of respondents, respectively. Somatostatin analogs (SSAs) were increasingly used as bridging strategy for delaying surgery (31%), and self‐injections or home care services were recommended by 35% and 48% of respondents, respectively. Overall, 63% of HPs declared that SSA self‐injections should be continued after the pandemic, although country‐based specificities are evident in this regard (UK, 94%; Germany, 40%; USA, 20%; *p* = .001). Treatment breaks of targeted therapies (17%), peptide receptor radionuclide therapy (13%) or chemotherapy (8%) were also proposed. When deemed necessary, surgery was preceded by testing for SARS‐CoV‐2 in 94% of cases. Surgical interventions were delayed due to lack of surgical provisions according to 40% of HPs. Multidisciplinary team meetings continued face‐to‐face similar to the pre‐pandemic period in 17% of cases, whereas virtual discussions were preferred by 77% of HPs. The frequency of meetings remained unmodified during the pandemic according to 58% of respondents. The majority of the HPs (59%) declared that the role of specialized nurses increased in importance during the pandemic.

### 3.3 COVID‐19‐induced changes in the management of NETs: the patient perspective

According to 56% of surveyed patients, the COVID‐19 pandemic added a substantial degree of complexity to their living with a NET. Appointments with NET specialists were cancelled or postponed in 21% of cases and changed to a remote appointment in 56% of cases. In particular, patients reported difficulties in accessing medical oncologists (45%), general practitioners (43%), endocrinologists/gastroenterologists (24%), general (28%) or specialist nurses (21%) and most inaccessible were surgeons (16%). When face‐to‐face consultations were performed, family members were not allowed to attend according to 42% of respondents. Although 81% of patients declared to understand such a need, a clear whish for family attendance in the future was expressed (always, 64%; on selected occasions, 33%) (Figure 1). No differences by stratifying factors (nationality, age, active treatment/surveillance status) were observed. Surgical interventions were cancelled or postponed based on 10% of responses, and this was related to patients' choice in 3% of cases. Delays in the access to laboratory tests or radiological investigations were reported by 22% and 30% of patients, respectively. Few patients (9%) chose to delay radiologic or endoscopic surveillance because they had been worried about the risk of potential infection with COVID‐19 within the hospital.

**FIGURE 2:**
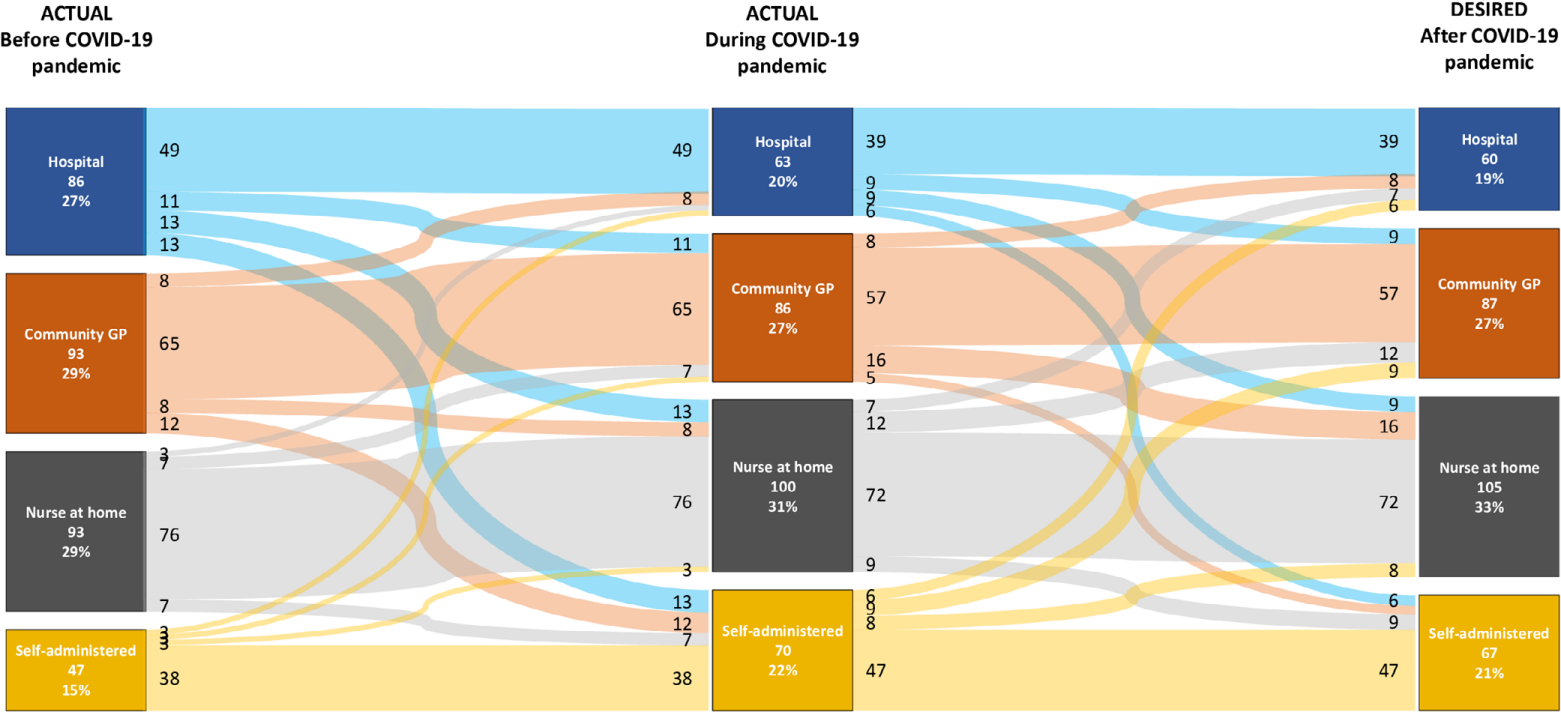
Somatostatin analog (SSA) injections settings: experiences and future preferences expressed by the surveyed patients. Sankey diagram shows changes in the setting of SSA administration before, during and after the COVID‐19 pandemic. An increase in home‐based administrations (self‐administration or professional administration at home) is coupled to a parallel decrease in community and, more markedly, hospital‐based administrations. No differences have been recorded by nationality, age group or treatment/surveillance status. GP, general practitioner

**FIGURE 3:**
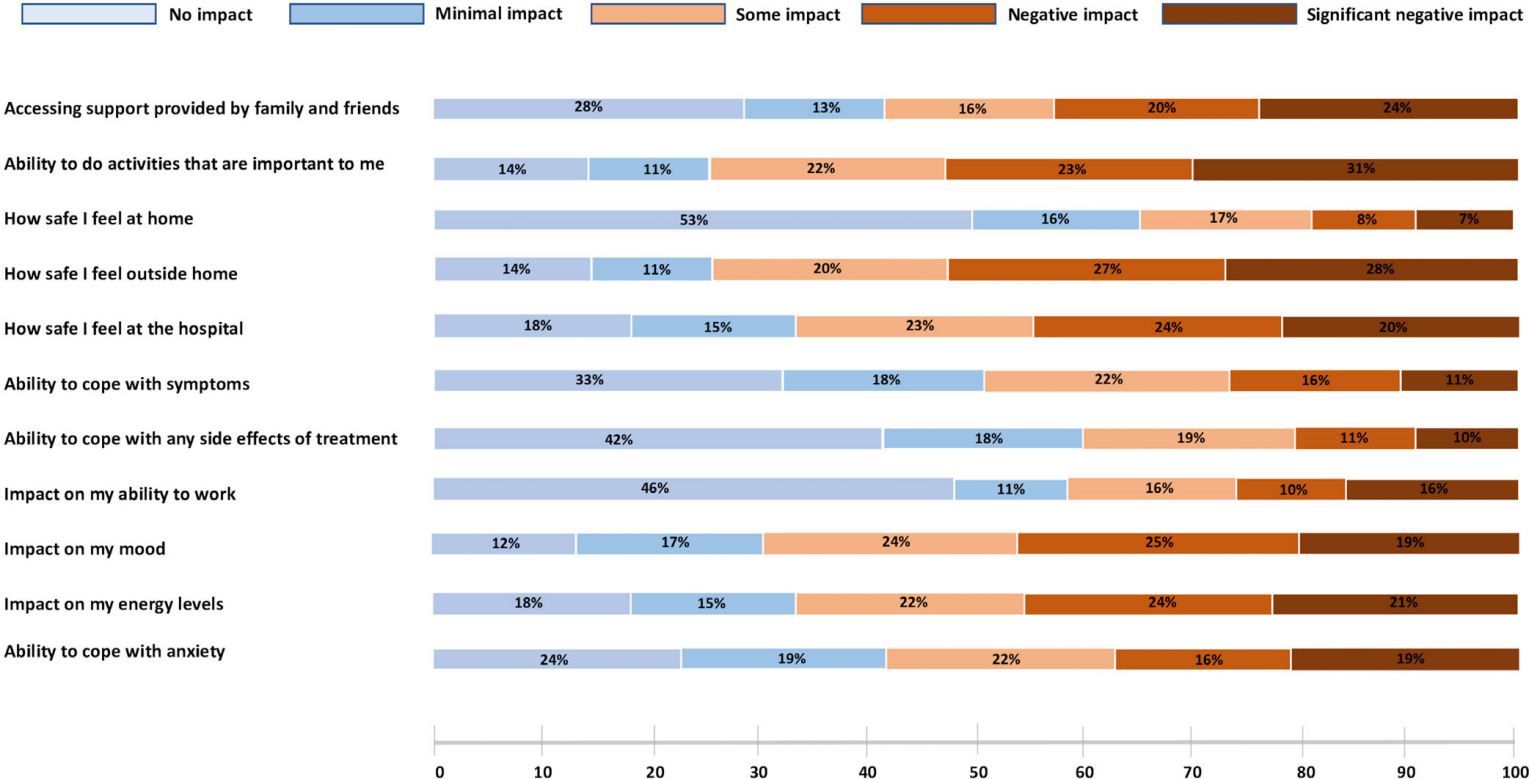
Impact of COVID‐19 on medical and non‐medical aspects important to neuroendocrine tumor (NET) patients. The bar graph shows how the pandemic increased the perception of uncertainty for activities to be performed outside home, negatively modulating a patients' mood and energy levels

The perceived impact of the pandemic on the oncological treatment was minimal, with 63% and 16% of patients denying treatment changes at all or reporting only modifications in the modality of administration of the treatment (i.e., self‐injections rather than hospital‐based injections). The oncological treatment was stopped, postponed, reduced in dosage or changed to a less immune suppressive one in 3%, 8%, 2% and 2% of cases, respectively. Difficulties in the access to the treatment were reported by a quarter of the responding patients, and 17% of the surveyed population declared that access to the care team administering injectable SSAs was negatively impacted by the pandemic. When comparing the modality of administration of SSAs before the pandemic, during the pandemic and in a foreseeable post‐pandemic scenario, a decrease in hospital‐based administrations and a parallel increase in home‐based administrations was noted, although more extensive changes could have been expected (Figure 2). In particular, the percentage of patients wishing to self‐administer SSAs or to receive SSAs at home by a nurse increased from 15% to 21% and from 29% to 33%, respectively, in the pre‐ and post‐pandemic scenario. Figure 3 provides an overview of the perceived impact of COVID‐19 pandemic on medical and non‐medical factors important to NET patients.

### 3.4 The role of anti‐SARS‐CoV‐2 vaccination in the management of NETs

Patients with resected NETs or advanced NETs were considered a priority group for vaccination by 19% and 93% of respondents, respectively. Only 8% and 4% of HPs considered changes in the treatment of patients with NETs before or after the COVID‐19 vaccination, respectively.

An anti‐SARS‐CoV‐2 vaccination was offered to and received by 95% and 91% of surveyed patients, respectively. The percentage of received vaccinations differed by countries, ranging from 95% to 98% in UK (98%), Canada (97%), Spain (96%), Ireland (96%) and Italy (95%), whereas it was substantially lower in USA, France and Germany (83%), as well as in Australia (76%). Concerns regarding the potential impact on the tumor were the main reason for the small minority declining the vaccination. The main source of patient information about the COVID‐19 vaccination was provided not only by physicians (51%), but also by local health authorities (10%), media (10%), patient advocacy groups (6%) and social media (5%). Notably, 43% of patients declared not to be sufficiently informed about the possible implications (if any) of the COVID‐19 vaccination on their NET disease.

### 3.5 The role of telemedicine in the present and future management of NETs

**FIGURE 4:**
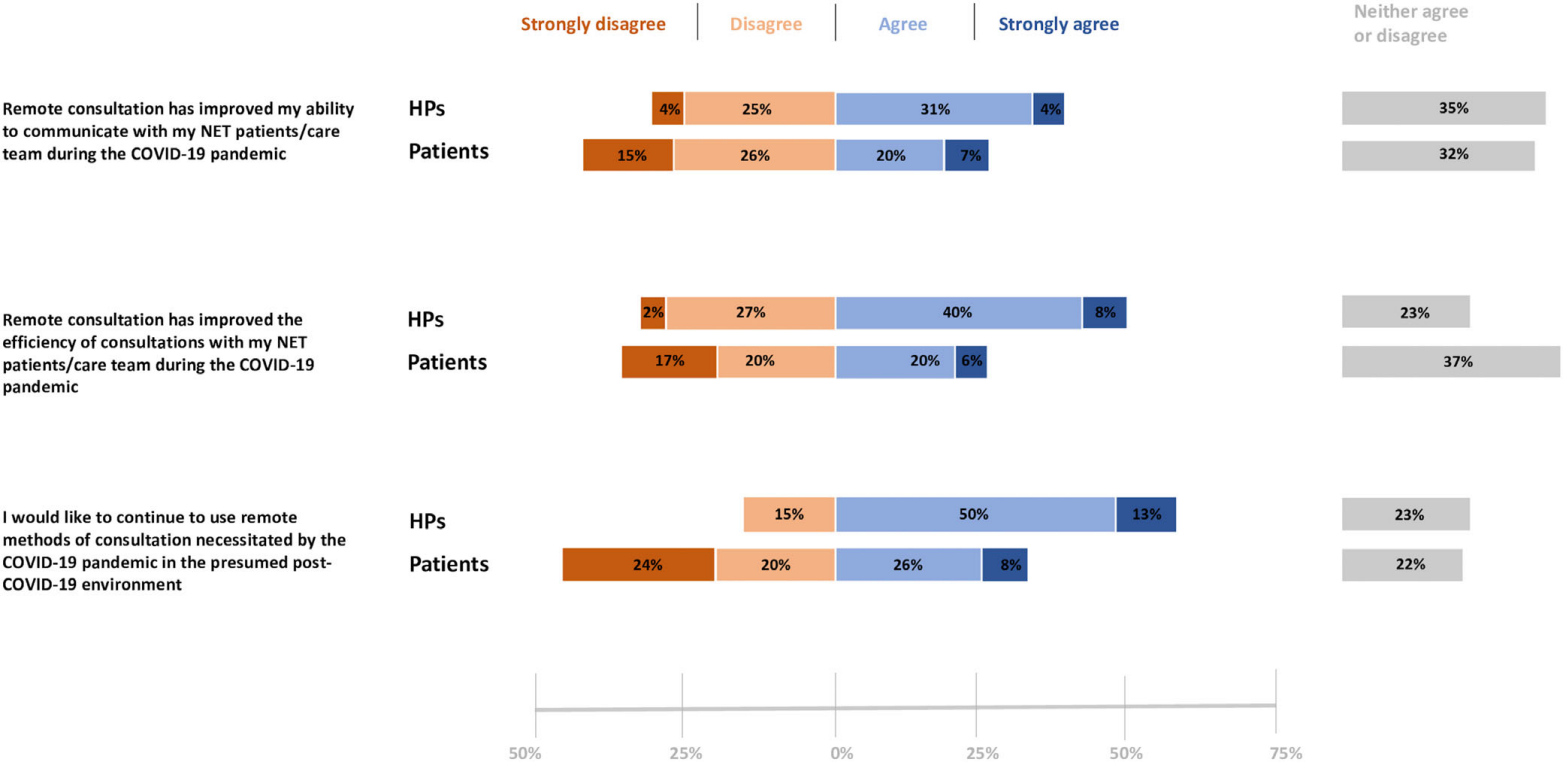
Patient and healthcare professional (HP) views on remote methods of consultations during and after the pandemic. The diverging stacked bar chart reveals differences in the perceptions of patients and healthcare professionals, particularly regarding the future implementation of telemedicine in a foreseeable post‐pandemic era

According to HP responses, the most employed remote methods used to consult with NET patients included phone calls (96%), emails (50%) and video consultations (44%). Only a minority of HPs (19%) used telemedicine apps. Virtual consultations were primarily used for post‐diagnosis discussion of treatment options (81%) and discussion of adverse events related to the treatment (79%). Diagnostic consults and discussion of supportive care measures were additional common topics for remote consultations (56% each). Overall, HPs had mixed views regarding the use of telemedicine, with 35% of them declaring that remote consultations improved their ability to communicate with NET patients, 30% stating the contrary and 35% being neutral (Figure 4). Nevertheless, 48% of HPs stated that remote consultations improved the efficiency of consulting with NET patients. Of note, a continued use of remote consultations in the post‐COVID scenario was envisioned by 63% of the HP respondents.

The 69% of the responding patients declared that remote methods of consultations were used more often during the pandemic than in the past. Telephone calls (77%), emails (40%) and video consultations (22%) were primarily used in this respect. Similarly to HPs, only a minority of patients (27%) reported an improved ability of communicating with the care team thanks to remote consultations, and only 26% of respondents found that the efficiency of consultations improved because of telemedicine (Figure 4). By contrast to the HP opinion, only 34% of patients declared a continued use of remote methods of consultations favorable in the presumed post‐pandemic era. However, age and nationality appeared to critically influence patients' views on this topic. Indeed, a sustained use of telemedicine was supported by 59%, 33% and 27% of patients < 41 years, 41–60 years and > 60 years respectively (*p* = .003), whereas positive opinions on the future use of telemedicine appeared to be less frequent among French or Spanish patients (24%) compared to US (52%) or German ones (80%) (*p* = .01). The main characteristics of patients in favor or against a continued use of telemedicine are summarized in Table 2.

**TABLE 2:** Profile of patients who wish or who do not wish a continued use of telemedicine in the foreseeable post‐pandemic era

	In favor^b^ of a continued use of telemedicine (*n* = 105)	Against^a^ a continued use of telemedicine (*n* = 135)	*p*
Gender, *n* (%)			.68
Female	69 (43)	93 (57)	
Male	36 (46)	42 (54)	
Age (years), *n* (%)			.04
< 40	20 (67)	10 (33)	
41–50	21 (47)	24 (53)	
51–60	34 (40)	51 (60)	
> 60	30 (37)	50 (63)	
Country, *n* (%)			.01
UK	15 (37)	26 (63)	
Germany	4 (80)	1 (20)	
France	4 (40)	6 (60)	
USA	13 (65)	7 (35)	
Italy	15 (68)	7 (32)	
Spain	10 (28)	26 (72)	
Ireland	6 (27)	16 (73)	
Australia	20 (44)	25 (56)	
Canada	11 (46)	13 (54)	
Years since NET diagnosis, *n* (%)			.68
< 2	17 (41)	24 (59)	
2–5	42 (40)	62 (60)	
6–10	23 (47)	26 (53)	
> 10	23 (50)	23 (50)	
Active treatment, *n* (%)			.48
Yes	92 (45)	114 (55)	
No	13 (38)	21 (62)	

*Note*: No response or neither agree or disagree have not been included in the analysis.

Abbreviation: NET, neuroendocrine tumor.

^a^ Disagree/strongly disagree.

^b^ Agree/strongly agree.

## 4 DISCUSSION

The COVID‐19 pandemic poses additional, extreme challenges on healthcare systems worldwide. It currently remains unclear how long the COVID‐19 outbreak will last, and the possibility that the disease might become endemic showing seasonal exacerbations cannot be excluded at present. Country‐specific interventions aimed at re‐organizing the healthcare systems in response to the COVID‐19 pandemic are underway, and healthcare providers, including those focused on NET care, need data and models that enable systematic, evidence‐based decisions. To improve strategies to mitigate the impact of COVID‐19 in the management of NETs, both patients’ and HPs’ opinions are key. In this work, we collected “real world” information on crucial aspects of current and future NET care paths through an international survey of both NET patients and HPs primarily dedicated to NET treatment. By doing so, we were able to detect differences and commonalities in the views expressed by the main stakeholders involved in the NET care process. Approximately half of the invited ENETS Centers of Excellence completed our survey. As result, although not being necessarily representative of the whole spectrum of Centers of Excellence, the findings of this study apply to institutions with a defined expertise in the management of NETs, and might not align with the views expressed in low‐volume centers.

Deferred diagnoses, delayed surveillance procedures and postponed elective surgeries are among the main negative consequences of the COVID‐19 outbreak according to our analysis. This appears to be similarly perceived by patients and HPs, and is in line with recent evidence from national surveys carried out in different phases of the pandemic (i.e., before and after the availability of vaccination).^15^, ^16^, ^17^ According to a large Italian multicenter analysis performed immediately after the first wave of the pandemic,^18^ the volume of pancreatic surgery decreased by approximately 20% after the onset of the COVID‐19 pandemic, and NETs were the least treated malignant disease (−34%). Although this is consistent with the most recent recommendations of the American College of Surgeons that consider most surgical interventions for NETs semi‐urgent (and therefore deferrable),^19^ different aspects should be taken into consideration when adapting health policy measures to the requirements superimposed by COVID in daily practice. First, given the possible persistence of the pandemic, the decrease of surgical volumes could potentially lead to the diagnosis of a higher rate of advanced stage diseases in the future. Second, delays in initiating treatment of patients well known to already experience long diagnostic delays,^20^ may have a profound psychological impact. In this regard, recent evidence has already shown a surge in depression and anxiety rates among NET patients subsequent to the initiation of the COVID‐19 outbreak.^21^ Therefore, although reallocation of healthcare resources has been necessary as an acute response to the pandemic, guaranteeing equitable access to care to all cancer patients irrespective of the evolution of the COVID‐19 outbreak is now of paramount importance.

Nevertheless, only limited changes to systemic treatments in response to the COVID‐19 pandemic have been reported by both patients and HPs in our analysis. A recommendation for home injection of SSAs was issued by more than 80% of surveyed HPs, and 53% of responding patients declared to have had the SSA administered at home by a nurse or self‐administered. Wide differences in the pattern of adoption of home injection (professional administration and self‐administration) were observed across countries, with the use of this strategy being low in USA (4%), Spain (12%) and Germany (17%), and high in Canada (64%), Italy (57%) and UK (50%).

Of note, before the pandemic a rather high number of patients (more than 40%) had received their SSA injections at home by a nurse, or had self‐administered SSAs. Thus the increase during the pandemic seems rather low. Approximately two‐thirds of HPs and half of patients would be in favor of a continued home‐based administration of SSAs. In this context, the impact of administration modality (self‐administration vs. professional administration) on patient outcomes is still under debate. In a recent analysis,^22^ patients who were self‐injecting SSAs had a higher risk of progression with respect to those who were administered by medical professionals, although these data need further confirmation.

The mental wellbeing of patients receiving care for their NETs during the COVID‐19 pandemic is of paramount importance. This survey shows that the COVID‐19 outbreak exerted a negative impact on a patient's mood, energy levels and ability to cope with anxiety, instilling a perception of unsafety for the activities performed outside the home. Time should be dedicated by treating physicians to inform patients on the known impact of COVID‐19 (and the relative vaccines) in the specific context of NET disease, and psychosocial support should be provided to relieve stress and anxiety. Recovery and restoration plans need to include strategies and infrastructures to address this increased need.

Another important aspect emerging from our survey is the perceived deterioration of the relationship between patients and HPs during the pandemic. This is likely to be the result of difficulties in access to NET specialists (in particular oncologists), cancelled or postponed appointments and the need to maintain contact by remote methods for review instead of face‐to‐face consultations as a result of pressure on the healthcare systems. Although the COVID‐19 outbreak has undoubtedly paved the way to the implementation of telemedicine in the care of patients with cancer, mixed views have been expressed by patients and HPs in this regard. Only a minority of patients and HPs have reported an improved ability to communicate through remote methods, whereas an improved efficiency of consulting was perceived by one‐half and one‐fourth of HPs and patients respectively. Strikingly, only one‐third of the responding patients was in favor of a continued use of telemedicine in a presumable post‐COVID scenario, whereas this proportion increased to two‐thirds based on the HP responses. Practical recommendations for the management of NET patients elaborated by leading NET specialists call for the use of virtual care and telemedicine whenever feasible during the COVID‐19 outbreak.^23^ However, the results of this survey emphasize the need for a tailored approach, with younger patients being more motivated to maintain the use of telemedicine. Although being key in connecting patients with their physicians, telemedicine should not increase the disparity between old and young patients or low‐income and high‐income patients. An area of safe and immediate implementation of telemedicine could be represented by multidisciplinary meetings, where virtual methods might increase the efficiency of work of participants, avoiding any risks of infection and delivering quicker decisions.

At the time of this survey, we recorded an extremely high rate of anti‐SARS‐CoV‐2 vaccination among interviewed subjects. No major treatment modifications in vaccinated patients were envisaged by HPs. This is probably a result of the manageable toxicity of most frequently used antitumor treatments in NET, such as SSAs. A need for better information around the anti‐SARS‐CoV‐2 vaccines and their implications on NETs emerges from the patient survey, and should be properly addressed by NET specialists, as well as national and international societies focusing on NETs.

Because the first wave of COVD‐19 changed our way of practicing medicine and communicating with cancer patients, new challenges have been raised, including vaccination and new COVID‐19 variants. With policy measures changing from local to international level every few months, it is fundamental to keep an eye on patients with low incidence neoplasms, including NET patients. Information achieved in surveys, including that of the present study, will help address the next steps in protecting NET patients, guaranteeing the highest standards of care, and adapting new resources such as telemedicine and home care services for SSA injection during unpredictable future times.

In conclusion, the spread of the COVID‐19 pandemic has exerted a negative impact on the management of NET patients, primarily determining diagnostic and surgical delays. With a hopeful but still unpredictable future regarding the pandemic, new health policy measures are needed to guarantee the highest standards of care for all patients with cancer. An individualized implementation of telemedicine in line with the needs of specific patient subpopulations should be favored, and self‐injection or home care services should be carefully weighed especially including the impact on patient mental wellbeing.

## AUTHOR CONTRIBUTIONS

**Mauro Cives:** Conceptualization; data curation; formal analysis; investigation; methodology; visualization; writing – original draft. **Jorge Hernando:** Conceptualization; data curation; methodology; writing – review and editing. **Angela Lamarca:** Conceptualization; investigation; methodology; writing – review and editing. **Catherine Bouvier:** Conceptualization; writing – review and editing. **Martyn Caplin:** Conceptualization; writing – review and editing. **Marianne Pavel:** Conceptualization; writing – review and editing.

## CONFLICTS OF INTEREST

Mauro Cives has received speakers’ fee from IPSEN and has consulted for AAA‐Novartis. Jorge Hernando has received speakers' fee from IPSEN, AAA‐Novartis, EISAI, Roche, Pfizer and Angelini. Angela Lamarca received travel and educational support from Ipsen, Pfizer, Bayer, AAA, SirtEx, Novartis, Mylan and Delcath; speaker honoraria from Merck, Pfizer, Ipsen, Incyte, AAA, QED, Servier, Astra Zeneca and EISAI; advisory and consultancy honoraria from EISAI, Nutricia Ipsen, QED, Roche, Servier, Boston Scientific, Albireo Pharma, AstraZeneca, Boehringer Ingelheim and GENFIT. Martyn Caplin has received speakers’ fee and research funding from IPSEN and AAA‐Novartis. Marianne Pavel has received speaker fees from AAA, IPSEN, Novartis, Amgen, Lilly and Boehringer Ingelheim, and fees for consultancy from AAA; IPSEN; Novartis, Hutchmed. INCA has received project funding from Ipsen, Novartis, AAA and ITM.

### PEER REVIEW

The peer review history for this article is available at https://publons.com/publon/10.1111/jne.13196.

## Supporting information


**Appendix S1** Supporting information

## Data Availability

Data are available upon request to the Authors.
