# Supplementary material for: The impact of COVID‐19 on the management of neuroendocrine tumors (NETS): An international NET CONNECT survey of NET patients and healthcare professionals treating net patients
Source: J Neuroendocrinol. 2022 Sep 7;34(10):e13196. doi: 10.1111/jne.13196 (PMC9786666; doi:10.1111/jne.13196)
Supplement: Supplementary file 1 — Appendix S1 Supporting information [file JNE-34-e13196-s001.docx]

**NETCOVID: Health Care Professional survey**

*NETCOVID is an international survey assessing the impact of the COVID-19 pandemic on people living with NET and HCPs managing the condition.*

*Analysis of anonymised results will go towards a proposed journal publication focused on the effects of the pandemic and lessons learned which may improve future care.*

*The survey will take no longer than 15 minutes to complete.*

**Entry/demographic questions**

*Please specify your country*

*Please specify your gender.*

-Male

-Female

Please specify your age.

- <30
- 31-40
- 41-50
- 51-60
- > 60

*Please specify your practice type and provide name of centre.*

- ENETS Centre of Excellence <<space for centre name to be added>>

- NET Centre of Expertise <<space for centre name to be added>>

- Other <<space for centre name to be added>>

*Which type of hospital do you work in?*

- University Hospital
- Public General Hospital
- Public Oncology Hospital
- Private Hospital
- Private Practice
- Other

Please describe your speciality.

- Endocrinologist
- Gastroenterologist
- Oncologist
- Pathologist
- Surgeon
- Nurse
- Other, please specify

Please indicate your years of experience in your discipline.

- < 2 years
- 2-5 years
- 6-10 years
- 11-20 years
- <20 years

*Please specify the number of patients with neuroendocrine tumours (NET) under your ongoing care.*

- < 50 patients
- 51-100 patients
- 101-250 patients
- 251 – 500 patients

- >500 patients

Have you tested positive with COVID-19? Yes/No

- - If yes, please provide month and year of positive COVID-19 test.
- *Have you been offered a COVID-19 vaccination?*

**Select one option**

- - Yes
  - No

*How many of your NET patients have tested positive for COVID-19?*

*How many of your NET patients have died from COVID-19 (based on local definition of a ‘COVID-related death’)?*

*Enter number below or select ‘Don’t know’ if number not known*

1. *Have you received/accessed tailored advice from professional societies or elsewhere on the treatment of patients with NET during the COVID-19 pandemic?*

- Yes
- No
- If yes, please specify the source:

1. Are there local country based COVID recommendations regarding the treatment of patients with NET where you practice?

- Yes
- No

2a. [If Q.2 = yes] To what extent are these recommendations being followed?

- Not at all followed
- Rarely followed
- Sometimes followed
- Usually followed

Always followed

1. Do you practice in an area of high COVID prevalence or high community spread?

- Yes
- No

[If yes] How has your own experience of COVID impacted your treatment decisions/ recommendations when treating patients with NET? [open-ended]

1. *To what extent do you agree or disagree with the statement below?
   The pandemic has* ***negatively affected*** *my ability to maintain a relationship with my patients:*

***Select one option***

- Strongly disagree
- Disagree
- Neither agree/nor disagree
- Agree
- Strongly agree

1. *How was your contact with patients living with NET managed during the pandemic?*

***Select one option***

- As usual in person
- As usual, but maintained via online consultations
- Reduced but partly maintained via mail/calls
- Only urgent visits
- Suspended

1. *Did you give / were you asked to give specific recommendations to patients with NET on how to deal with the pandemic?*

***Select all relevant options***

- Patients asked if they were at increased risk from COVID-19 related complications
- Patients asked how to manage their disease during the pandemic
- Patients asked how to manage their medical therapy during the pandemic
- During the consultations that took place during the pandemic, we advised on general health measures
- During the consultations that took place during the pandemic, we instructed patients on their disease management

1. *In my practice the COVID-19 pandemic* ***has negatively affected:***

***Select all relevant options***

- The NET diagnostic pathway
- Patients presenting with worsening symptoms
- Healthcare professional’s capacity to assess patients
- Laboratory/pathology capacity to process samples and conduct tests
- Radiologist capacity for imaging diagnostics
- Ongoing NET treatment
- None of the above

1. *How has locoregional therapy for NET been affected in your practice during the COVID pandemic?*

***Select all relevant options***

- Increased use of ‘watch and wait’ (surveillance)
- Delayed frequency of surveillance (i.e. endoscopic surveillance)
- Delayed surgical resection/debulking surgery
- Started pre-operative SSA as a bridging strategy in patients with delayed surgery
- Postponed Liver directed therapy
- Recommended stereotactic body radiotherapy in place of liver directed therapy or as a bridge for delayed surgery
- Delayed palliative radiotherapy
- None of the above

1. *In patients treated with SSAs, which changes to therapy have you made during the COVID-19 pandemic?*

***Select all relevant options***

- Recommended treatment delay or interruption to avoid potential patient exposure to virus
- Increased the interval between injections of somatostatin analogues
- Increased doses of somatostatin analogue injections or increased the frequency of injections
- Recommended self-injection of somatostatin analogues
- Recommended home injection service
- None of the above

1. *What changes* *have you made to other systemic therapies during the COVID-19 pandemic?*

***Select all relevant options***

- Reduced the dose or recommended a treatment break from targeted therapies (everolimus/sunitinib)
- Omitted a cycle or extended an interval between PRRT treatments
- Delayed or interrupted chemotherapy
- Added interferon-alpha to SSAs for proactive functional control to avoid hospitalisations
- Added telotristat ethyl to SSAs for proactive functional control to avoid hospitalisations
- None of the above

1. *To what extent do you agree or disagree with the statement below?*

*The role of self injections of* somatostatin analogues *has become more important during the COVID-19 pandemic.*

***Select one option***

- Strongly disagree
- Disagree
- Neither agree/nor disagree
- Agree
- Strongly agree

1. *I plan for the patients who have started to self-inject during the pandemic, to continue with self-injection after the pandemic.*

***Select one option***

- Strongly disagree
- Disagree
- Neither agree/nor disagree
- Agree
- Strongly agree

1. *To what extent do you agree or disagree with the statement below?
   A lack of personal protective equipment has reduced my ability to treat patients with NET.*

***Select one option***

- Strongly disagree
- Disagree
- Neither agree/nor disagree
- Agree
- Strongly agree

1. *In cases of where surgery was intended have you?*

***Please select yes or no for each option***

- Carried out a COVID-19 test prior to surgery
- Delayed surgery due to lack of testing provision
- Delayed surgery due to lack of surgical provision
- Not carried out pre-surgical testing due to low infection levels in your area

1. *To what extent do you agree or disagree with the statement below?
   Patients with resected NET should be considered a priority group for COVID vaccination.*

***Select one option***

- Strongly disagree
- Disagree
- Neither agree/nor disagree
- Agree
- Strongly agree

1. *To what extent do you agree or disagree with the statement below?
   Patients with advanced NET should be considered a priority group for COVID vaccination.*

***Select one option***

- Strongly disagree
- Disagree
- Neither agree/nor disagree
- Agree
- Strongly agree

1. *Would you consider changing the treatment of a patient living with NET* ***before*** *they receive a vaccination for COVID-19?*

***Select one option***

- Yes
- No

*If yes, which treatments would be affected and why?*

***Select all relevant options***

- Somatostatin analogues
- Targeted therapies (everolimus/sunitinib)
- PRRT
- Chemotherapy
- Interferon-alpha
- Telotristat ethyl

*Please provide an explanation as to your response above:*

1. *Would you consider changing the treatment of a patient living with NET* ***after*** *they receive a vaccination for COVID-19?*

***Select one option***

- Yes
- No

*If yes, which treatments would be affected and why?*

***Select all relevant options***

- Somatostatin analogues
- Targeted therapies (everolimus/sunitinib)
- PRRT
- Chemotherapy
- Interferon-alpha
- Telotristat ethyl

*Please provide an explanation as to your response above:*

1. *Did you allow your NET patients to have a family member accompany them for a face-to-face clinic/hospital visit during the COVID-19 pandemic?*

**Select one option**

- - Yes, at every visit
  - Only on some selected occasions (when the patient requested it or if considered to be required by the treating team)
  - No never, the patient attended all *face-to-face* consultations on their own
  - I have not had any *face-to-face* consultations

1. *If your NET patients attended face-to-face consultations on their own during the COVID-19 pandemic, how do you think they felt?*

**Select one option**

- - They seemed to feel comfortable and appeared to understand the need
  - They seemed anxious but appeared to understand the need
  - They seemed overwhelmed and felt that their family should have been allowed
  - Other

1. *For future face-to-face appointments during the COVID-19 pandemic what is your view about family attendance:*

**Select one option**

- - Family members should always be allowed
  - Family members should only be allowed on specific occasions
  - Family members should never be allowed

1. *What were the requirements for outpatient visits at your centre?*

**Select all that apply**

- - Patient was triaged for COVID related symptoms first (eg. temperature taken and asked about potential symptoms)
  - Patient had to have a negative COVID test prior to attending the clinic/hospital
  - No specific requirements other than the usual COVID-19 practice of face masks, social distancing rules and handwashing

1. *Which remote methods have you used to consult with people with NET?*

***Select all relevant options***

- Telephone consultation
- Video consultation (Skype, Zoom, etc.)
- Telemedicine apps (MDLIVE, Live Health etc.)
- E-mail
- I have not used remote methods for consultation

1. *Which types of consultation have you carried out virtually with people with NET?*

***Select all relevant options***

- Diagnostic consult
- Post-diagnosis discussion of condition and treatment options
- Training of patient/family members on administration of somatostatin analogues at home
- Supportive care (eg. nutrition, psychological support)
- Discussion of alternative treatment options due to the effect of the pandemic
- Discussion of adverse events related to treatment

1. *In what way has the COVID-19 pandemic affected your multidisciplinary team (MDT) meetings?*

***Select all relevant options***

- MDT meetings continued face to face/in the same way as pre-pandemic
- Multidisciplinary discussion with other HCPs/referral centres occurred virtually
- MDT meetings were held according to the usual schedule and frequency of meetings
- The frequency of MDT meetings was reduced during the COVID pandemic
- MDT meetings were held but with just one representative for each discipline (reduced number)

1. *To what extent do you agree or disagree with the statement below?
   Remote consultation has improved my ability to communicate with my patients living with NET during the COVID-19 pandemic:*

***Select one option***

- Strongly disagree
- Disagree
- Neither agree/nor disagree
- Agree
- Strongly agree

1. *To what extent do you agree or disagree with the statement below?
   Remote consultation has improved the efficiency of consultations with my patients living with NET during the COVID-19 pandemic:*

***Select one option***

- Strongly disagree
- Disagree
- Neither agree/nor disagree
- Agree
- Strongly agree

1. *To what extent do you agree or disagree with the statement below?
   I will continue to use remote methods of consultation necessitated by the COVID-19 pandemic in the presumed post-COVID-19 environment:*

***Select one option***

- Strongly disagree
- Disagree
- Neither agree/nor disagree
- Agree
- Strongly agree

1. *To what extent do you agree or disagree with the statement below?*

*During the pandemic the role of specialised nurses has increased in importance:*

***Select one option***

- Strongly disagree
- Disagree
- Neither agree/nor disagree
- Agree
- Strongly agree

1. *Please add any thoughts you have in the below dialog box, in particular relating to changes in practice which you will continue to use in the future, and challenges/opportunities not covered in the above survey (open ended).*
2. *Do you feel the COVID vaccination is going to change your current practice – free text*

NETCOVID Patient survey

**Please note this survey is intended for patients with advanced neuroendocrine disease, and not for patients in follow up after cure of a neuroendocrine tumour.**The purpose of the survey is to gather anonymous information on how the COVID-19 pandemic has impacted your daily life, your access to treatment and/or testing, and your visits to a nurse or specialist.
The survey will take no longer than 10 minutes to complete. Your participation will help all healthcare practitioners involved in the management of NET better understand how crucial continuous support and access to medicines are for patients with a chronic rare disease during the pandemic.
Your individual responses will be grouped with all other responses. Please be assured that your identity will be protected at all times.
Thank you for your time.

SA Please select your country.

SA1 Please specify your country.

________________________________________________________________

SA2 In which state do you currently practice?

S1 Please specify your gender.

- Male
- Female
- Other (please specify): ________________________________________________

S2 Please specify your age.

- <31
- 31 – 40
- 41 – 50
- 51 – 60
- 61-70
- > 70

S3 Number of years since diagnosis with neuroendocrine tumour (NET)?

- <2 years
- 2-3 years
- 4-5 years
- 6-10 years
- 11-15 years
- >15 years

S4a Are you on any treatment for your neuroendocrine tumour?

- Yes
- No

S4b Which treatment(s)?

- Lanreotide / octreotide (somatostatin analogue injections)
- Everolimus / sunitinib tablets (targeted therapies)
- Lutetium therapy (PRRT)
- Oral chemotherapy
- Intravenous chemotherapy
- Interferon injections
- MIBG
- Other (please specify): ________________________________________________

S5 Have you **tested positive** with COVID-19?   *(This question is optional)*

- Yes
- No

S5 Please provide the date of your positive COVID-19 test.

S6a Are you member of a NET patients association / patients advocacy group?

- Yes
- No

S6b Which ones?

________________________________________________________________

________________________________________________________________

________________________________________________________________

________________________________________________________________

________________________________________________________________

**Please read the statement below and select the response that best reflects your experience living with NET during the COVID-19 pandemic.**

Q1 The COVID-19 pandemic has made it more challenging than normal to live with NET.

- Strongly disagree
- Disagree
- Neither agree nor disagree
- Agree
- Strongly agree

Q2 How has your care been affected during the COVID-19 pandemic?

(*Select all of the relevant options*)

- My appointment with a specialist was cancelled/postponed
- My appointment with a specialist was changed to a remote appointment
- My surgery was cancelled/postponed
- I chose to postpone my surgery because I was worried about COVID-19 infection
- I was unable to access the hospital for blood tests (
- I was unable to access the scans or other investigations
- I chose to delay my scans/endoscopic surveillance because I was worried about COVID-19 infection
- My usual care team were deployed into other areas (e.g., intensive care)
- I was not able to access other health and social care services important to me both in the hospital and in the community
- Other (please specify): ________________________________________________

|  |
| --- |

Q3 How has your treatment been affected during the COVID-19 pandemic?


(*Select all of the relevant options*)

- My medical/oncological treatment was stopped because of the COVID-19 pandemic
- My medical/ oncological treatment was postponed because of the COVID-19 pandemic
- My medical/ oncological treatment was changed to a less immune suppressive one
- The dose of my medication was reduced
- My treatment went ahead as usual with alternative ways of me receiving my treatment (e.g. self-injection or postal delivery of treatment)
- I chose to delay my treatment because I was worried about COVID-19 infection
- A limit was set on the amount of medicines I could collect/order compared to before the pandemic
- I was given a larger supply / prescriptions of my treatments to last longer between clinic visits
- Other (please specify): ________________________________________________
- *None - My treatment has not been affected at all by the COVID-19 pandemic*

Q4 To what extent do you agree or disagree with the statement below **for each healthcare team member?**


 *The COVID-19 pandemic has* ***negatively impacted*** *my ability to access these members of my healthcare team:

 (Please rate your level of agreement for each option*)

|  | Strongly  disagree | Disagree | Neither agree  nor disagree | Agree | Strongly  agree | Not  relevant |
| --- | --- | --- | --- | --- | --- | --- |
| Endocrinologist / Gastroenterologist |  |  |  |  |  |  |
| Pulmonologist |  |  |  |  |  |  |
| Medical Oncologist |  |  |  |  |  |  |
| Specialist nurse |  |  |  |  |  |  |
| General nurse |  |  |  |  |  |  |
| Primary care physician/General Practitioner |  |  |  |  |  |  |
| Pharmacist |  |  |  |  |  |  |
| Surgeon |  |  |  |  |  |  |
| Other (please specify): |  |  |  |  |  |  |

Q5 The remote methods I have used to have contact with my care team during the COVID-19 pandemic are: *(Select all relevant options)*

- Telephone consultations
- Video consultations
- Telemedicine app
- Emailing
- I have not used any remote methods/channels to interact with my care team

**To what extent do you agree or disagree with the statements below?**

Q6 During the pandemic I have met with my care team using remote methods (e.g. telephone/video consultation etc.) **more often** than in the past.

- Strongly disagree
- Disagree
- Neither agree nor disagree
- Agree
- Strongly agree

Q7 Remote consultation has improved my ability to communicate with my care team during the COVID-19 pandemic.

- Strongly disagree
- Disagree
- Neither agree nor disagree
- Agree
- Strongly agree

Q8 Remote consultation has improved the efficiency of consultations with my care team during the COVID-19 pandemic.

- Strongly disagree
- Disagree
- Neither agree nor disagree
- Agree
- Strongly agree

Q9 I would like to continue to use remote methods of consultation necessitated by the COVID-19 pandemic in the presumed post-COVID-19 environment.

- Strongly disagree
- Disagree
- Neither agree nor disagree
- Agree
- Strongly agree

**To what extent do you agree or disagree with the statements below?**

Q10 The COVID-19 pandemic has **negatively impacted** my ability to access my treatment for NET.

- Strongly disagree
- Disagree
- Neither agree nor disagree
- Agree
- Strongly agree

Q11 The COVID-19 pandemic has **negatively impacted** my ability to access the care team that would normally administer injectable therapies [such as somatostatin analogue injections (lanreotide/octreotide)].

- Strongly disagree
- Disagree
- Neither agree nor disagree
- Agree
- Strongly agree
- Not relevant / Not applicable

Q12 During the COVID-19 pandemic I have:
(*Select all relevant options*)

- Sought advice regarding self or partner administered somatostatin analogue (SSA) injections for my NET
- Been advised to switch to self or partner administered SSA injections for my NET
- Switched to self or partner administered SSA injections for my NET
- Been unable to access self or partner administered SSA injections for my NET
- Continued with my injectable SSA therapy as normal for my NET
- Decided to stop injectable SSA therapy for my NET
- *None of the above*

Q13 Before the COVID-19 pandemic, I was receiving my SSA injections:


(*Select all relevant options*)

- In the hospital
- In the community or with my General Practitioner
- At home by a nurse
- Self-administered or partner administered

Q14 During the COVID-19 pandemic, I was receiving my SSA injections:


(*Select all relevant options*)

- In the hospital
- In the community or with my General Practitioner
- At home by a nurse
- Self-administered or partner administered

Q15 After the COVID-19 pandemic, I would like to receive my SSA injections:

- In the hospital
- In the community or with my General Practitioner
- At home by a nurse
- Self-administered or partner administered

**To what extent do you agree or disagree with the statements below?**

Q16 The COVID-19 pandemic has **negatively impacted** my ability to provide blood and urine samples for regular lab testing.

- Strongly disagree
- Disagree
- Neither agree nor disagree
- Agree
- Strongly agree
- Not relevant

Q17 The COVID-19 pandemic has **negatively impacted** my ability to have my scans in the normal timeframe

- Strongly disagree
- Disagree
- Neither agree nor disagree
- Agree
- Strongly agree
- Not relevant

Q18 To what extent has the COVID-19 pandemic **negatively impacted** other important factors in your life?

*(where 1 = no / minimal impact and 5 = significant negative impact)*

|  | No /  minimal  impact  1 | 2 | 3 | 4 | Significant  negative  impact  5 |
| --- | --- | --- | --- | --- | --- |
| Accessing support provided by family and friends |  |  |  |  |  |
| Ability to do activities that are important to me |  |  |  |  |  |
| How safe I feel at home |  |  |  |  |  |
| How safe I feel outside home |  |  |  |  |  |
| How safe I feel at the hospital |  |  |  |  |  |
| Ability to cope with symptom |  |  |  |  |  |
| Ability to cope with any side effects of treatment |  |  |  |  |  |
| Impact on my ability to work |  |  |  |  |  |
| Impact on my mood |  |  |  |  |  |
| Impact on my energy levels |  |  |  |  |  |
| Ability to cope with anxiety (due to risks, lack of sufficient medical treatment etc) |  |  |  |  |  |

Q19 Were you allowed to have a family member accompany you for a face-to-face clinic/hospital visit during the COVID-19 pandemic?

- Yes, at every visit
- Only on some selected occasions (when I requested it or when my treating team felt it was necessary)
- No never, I have attended all face-to-face consultations on my own
- I have not had any face-to-face consultations

Q20 If you have attended face-to-face consultations on your own during the COVID-19 pandemic, how did you feel?

- I felt comfortable and I understood the need
- I felt anxious but I understood the need
- I felt overwhelmed and I feel my family should have been allowed
- Other (please specify) ________________________________________________

Q21 For future face-to-face appointments during the COVID-19 pandemic, what is your view about family attendance?

- Family members should always be allowed
- Family members should only be allowed on specific occasions
- Family members should never be allowed

Q22 **To what extent do you agree or disagree with the statement below?**

The COVID-19 pandemic has **negatively impacted** my/my family’s financial situation.

- Strongly disagree
- Disagree
- Neither agree nor disagree
- Agree
- Strongly agree
- Not relevant

Q23 Have you been offered a COVID-19 vaccination?

- Yes
- No

Q24 Have you received a COVID-19 vaccination at the time of completing this survey?

- Yes
- No

Q24a Why have you not received a COVID-19 vaccination, even though you have been offered one?

- Awaiting appointment
- I have declined the vaccination due to concerns regarding the potential impact on treatment for my neuroendocrine disease
- I have declined the vaccination for other reasons

Q25 Did you feel sufficiently informed about the COVID-19 vaccination and any implications for your neuroendocrine disease?

- Yes
- No

Q25a What was your main source of information about the COVID-19 vaccination and any implications for your neuroendocrine disease?

- Your physician
- Local Health authority letter/information
- Media (i.e., news/newspapers)
- Social media
- Other (please specify): ________________________________________________

|  |
| --- |

Q26 During the COVID-19 pandemic, I have accessed the following sources of support/information on NET **more often** than in the past:

- National/international patient advocacy groups
- Local support groups
- Facebook communities
- Medical websites (e.g. WebMD, BMJ, hospital websites)
- Pharmaceutical companies’ websites/medical information services
- Other (please specify): ________________________________________________

Q27 What changes to your care would you recommend to others and/or like to continue to use after the pandemic?

________________________________________________________________

________________________________________________________________

________________________________________________________________

________________________________________________________________

________________________________________________________________

Q28 Please use the below space to describe any impacts the COVID-19 pandemic has had on your life or care that are not covered above.

________________________________________________________________

________________________________________________________________

________________________________________________________________

________________________________________________________________

________________________________________________________________

Q29 Please use this space to share any further thoughts or feelings.

________________________________________________________________

________________________________________________________________

________________________________________________________________

________________________________________________________________

________________________________________________________________

[APPENDIX]

**FULL LIST OF COUNTRIES**

Argentina

Armenia

Australia

Austria

Bangladesh

Belarus

Belgium

Brazil

Bulgaria

Cambodia

Cameroon

Canada

Chile

China

Colombia

Costa Rica

Croatia

Czech Republic

Denmark

Ecuador

Egypt

Estonia

Finland

France

Georgia

Germany

Ghana

Gibraltar

Greece

Greenland

Guatemala

Hong Kong

Hungary

Iceland

India

Indonesia

Iran

Iraq

Ireland

Israel

Italy

Japan

Jordan

Kenya

Kuwait

Laos

Latvia

Liechtenstein

Lithuania

Luxembourg

Macedonia

Malaysia

Mexico

Mongolia

Montenegro

Morocco

Netherlands

New Zealand

Nicaragua

Nigeria

Norway

Oman

Pakistan

Panama

Peru

Philippines

Poland

Portugal

Qatar

Romania

Russia

Saudi Arabia

Senegal

Serbia and Montenegro

Singapore

Slovakia

Slovenia

South Africa

South Korea

Spain

Sri Lanka

Sweden

Switzerland

Taiwan

Tajikistan

Thailand

Turkey

Turkmenistan

Ukraine

United Arab Emirates

United Kingdom

United States

Uruguay

Uzbekistan

Venezuela

Vietnam

Yugoslavia

Other (please specify): ______
